# Integrated Ultrasonication and Microbubble-Assisted Enzymatic Synthesis of Fructooligosaccharides from Brown Sugar

**DOI:** 10.3390/foods9121833

**Published:** 2020-12-10

**Authors:** Worraprat Chaisuwan, Apisit Manassa, Yuthana Phimolsiripol, Kittisak Jantanasakulwong, Thanongsak Chaiyaso, Wasu Pathom-aree, SangGuan You, Phisit Seesuriyachan

**Affiliations:** 1 Interdisciplinary Program in Biotechnology, Graduate School, Chiang Mai University, Chiang Mai 50200, Thailand; worraprat_chai@cmu.ac.th (W.C.); apisit_man@cmu.ac.th (A.M.); 2 Faculty of Agro-Industry, Chiang Mai University, 155 Moo 2, Mae Hia, Mueang, Chiang Mai 50100, Thailand; yuthana.p@cmu.ac.th (Y.P.); kittisak.jan@cmu.ac.th (K.J.); thanongsak.c@cmu.ac.th (T.C.); 3 Cluster of Agro Bio-Circular-Green Industry (Agro BCG), Chiang Mai University, Chiang Mai 50200, Thailand; 4 Department of Biology, Faculty of Science, Chiang Mai University, Chiang Mai 50200, Thailand; wasu.p@cmu.ac.th; 5 Department of Marine Food Science and Technology, Gangneung-Wonju National University, Gangneung, Gangwon 210-702, Korea; umyousg@gwnu.ac.kr

**Keywords:** fructooligosaccharide, optimisation, Box–Behnken, ultrasound, microbubble, transfructosylation, enzyme activity enhancement

## Abstract

Fructooligosaccharides (FOS) are considered prebiotics and have been widely used in various food industries as additives. Ultrasonication has been widely used to enhance food processes; however, there are few reports on ultrasound-assisted FOS synthesis. In the present study, FOS were produced from brown sugar using ultrasonication combined with microbubbles, and the production was optimised using a Box-Behnken experimental design. Here we showed that a combination of ultrasonication and microbubbles could boost the enzyme activity by 366%, and the reaction time was shortened by 60%. The reaction time was a significant variable affecting the FOS production. The optimum conditions were 5 min 45 s of ultrasonication and 7 min 19 s of microbubbles with a reaction time of 5 h 40 min. The maximum enzyme activity and total FOS yield were 102.51 ± 4.69 U·mL^−1^ and 494.89 ± 19.98 mg·g^−1^ substrate, respectively. In an enlarged production scale up to 5 L, FOS yields were slightly decreased, but the reaction time was decreased to 4 h. Hence, this technique offers a simple and useful tool for enhancing enzyme activity and reducing reaction time. We have developed a pilot technique as a convenient starting point for enhancing enzyme activity of oligosaccharide production from brown sugar.

## 1. Introduction

Nowadays, people have attempted to improve their health and diet due to increased non-communicable diseases, including cancers, diabetes, metabolic syndrome, hypertension, stroke, and heart disease [1]. Therefore, many products, such as functional foods that have positive effects on health have been developed. Functional foods can be defined as foods that provide nutrients and energy, modulate an individual’s health and physiological functions, and reduce diseases [2]. Oligosaccharides (short-chain carbohydrates) with prebiotic properties such as fructooligosaccharides (FOS), galactooligosaccharides (GOS), xylooligosaccharides (XOS), inulooligosaccharides (IOS), soybean oligosaccharides (SOS), and cello-oligosaccharides (COS) have been interested from researchers because they have potential as ingredients in functional foods [3,4]. Among them, FOS are well-known non-digestible carbohydrates and have been extensively used in various food products [5].

It is safe to use FOS in food products, since FOS have been recognised by the Food and Drug Administration of the United States to be generally safe (GRAS) [6,7]. In the food industry, because FOS have favourable characteristics, including low calories, sweetening property, and unique physiochemical properties, they have been used as food additives in pastry, confectionery and dairy products [8,9]. Furthermore, FOS have many advantages for an individuals’ health due to their biological activities, including prebiotic, anticancer, and immunomodulatory properties. FOS serve as carbon and energy sources for probiotics in the large intestine. FOS can promote the growth of prebiotic strains such as *Bifidobacterium* spp. and *Anaerostipes caccae*, butyrate-producing bacterium [10,11]. In contrast, FOS can inhibit the growth of some pathogenic bacteria such as *Clostridium* spp. [12]. As a prebiotic effect, gut microbiota can ferment FOS and then produce short-chain fatty acids (SCFAs), such as acetate, butyrate and propionate, which modulate intestinal epithelial functions and activate the host’s immunity [13]. Buddington and colleagues [14] reported that FOS-fed mice had a low number of *Listeria monocytogenes* and *Salmonella enterica* subsp. *enterica* serovar Typhimurium (pathogenic bacteria). Taper and Roberfroid [15] also reported that FOS exhibited anti-proliferative effects on cancer cells in mice. However, consuming high levels of FOS (>20 g/day) might cause abdominal distension, abdominal rumbling, abnormal flatulence and abdominal pain because of excess gases from bacterial metabolism in the large intestine [16].

FOS are carbohydrates consisting of fructose monomers (2–10 residues) and can be naturally found in plant sources, such as onion, barley, asparagus, garlic, banana, and Jerusalem artichoke [17,18]. Moreover, FOS can be synthesised from low-cost materials such as sucrose and longan syrup, using an enzymatic reaction of transfructosylating enzymes, also called transfructosylation [19]. Enzymes with transfructosylation activity can transfer fructosyl groups, which are significant in FOS synthesis [20]. In the enzymatic synthesis, FOS can be produced using various enzymes, including *β*-fructofuranosidases (EC 3.2.1.26), *β*-fructosyltransferases (EC 2.4.1.9), and inulinases (EC 3.2.1.7) [8,20,21]. The commercial enzyme Pectinex Ultra SP-L was derived from *Aspergillus aculeatus* and is a complex enzyme, mainly consisting of polygalacturonase (pectinase) and others (cellulase, *β*-galactosidase and *β*-fructosyltransferases). This enzyme has been widely used for FOS production from sugar because it possesses high fructosyltransferase activity and was used in the present study [19,22]. After FOS synthesis, FOS molecules are a mixture of 1-kestose (1-kestotriose; GF2), nystose (1,1-ketstotetraose; GF3), and 1^F^-*β*-fructofuranosylnystose (1,1,1-kestopentaose; GF4), which are categorised by the number of fructose monomers linking to glucose [8]. After production, some glucose and fructose contents remain as by-products, as well as excess sucrose, so it is necessary to purify the product. There are various techniques for purification of FOS, for example, chromatography, membrane filtration (such as nanofiltration), charcoal column and so on [23,24,25]. 

Various chemical, physical and biological approaches have been used to enhance enzyme activity for the improvement of FOS production. Among the physical methods, ultra-high pressure (UHP) is a useful technique that can activate or inactivate enzyme activity. In optimal levels of pressure, UHP enhanced fructosyltransferase activity of Pectinex Ultra SP-L and FOS contents could be increased 2.5-fold [5].

In the last few decades, an ultrasonication has been widely applied in various food and biotechnological processes. This technique has been performed as an enhancement for metabolite extraction from natural sources, such as fruits, microbial cells, and vegetables, among others, and can change the structure of food products [26,27]. This method has been used for enzyme inactivation; however, a large number of reports have been confirmed that the technique was able to activate some enzymes under suitable conditions [28]. Additionally, an ultrasonication has been approved that can increase enzyme activity at an appropriate frequency and intensity levels because ultrasound can act on conformation changes in enzymes or substrates [28,29]. Ultrasound can improve the activity of many enzymes including *α*-amylase, *β*-d-glucosidase, cellulose, dextranase and lipase [28,30,31,32]. Therefore, this method has been extensively used in various food industries, especially in fruit juice production, where it slightly affects the quality of fruit juice [33,34]. However, there are few reports on the use of ultrasonication for FOS production. This study, therefore, aimed to investigate the effects of ultrasonication and microbubble on enhancing enzyme activity for FOS production. Moreover, optimisation of the processes was done by a Box-Behnken design, and FOS yields (mg·g^−1^ substrate) were quantified using high-performance liquid chromatography (HPLC).

## 2. Materials and Methods 

### 2.1. Enzymes and Chemicals

Pectinex Ultra SP-L, a commercial pectinolytic and cellulolytic enzyme derived from *Aspergillus aculeatus*, was purchased from Novozymes (Bagsværd, Denmark). Commercial brown sugar and white sugar derived from sugar canes were used as substrates in this study. 3,5-dinitrosalicylic acid (DNS) was purchased from Sigma-Aldrich (St. Louis, MO, USA). Standard carbohydrates including 1-kestose, nystose, 1^F^-*β*-fructofuranosylnystose, sucrose, glucose and fructose were obtained from Wako Pure Chemical Industries (Osaka, Japan).

### 2.2. Enzymatic Assay

Enzyme activity was determined based on the generation of reducing sugar using sucrose as a substrate [19]. A concentration of Pectinex Ultra SP-L used in this study was 100 U·mL^–1^, which was reported in a previous study [5]. Briefly, sucrose was dissolved in a sodium acetate buffer (pH 6.5) at a final concentration of 2% (*w/v*). Next, 0.2 mL of Pectinex Ultra SP-L and 1.8 mL of sucrose solution were mixed and then incubated at 55 °C for 15 min. Reducing sugars released from sucrose were analysed using the DNS assay [35]. After incubation, the sample (1 mL) and DNS reagent (4 mL) were mixed in a test tube and the test tube was placed in a boiling water bath for 5 min. The tube was placed in ice to instantly cool and then laid in a room temperature water bath (25 °C). Finally, the absorbance was measured at 540 nm using a Genesys 20 spectrophotometer (Thermo Fisher Scientific Inc., Waltham, MA, USA). One unit of Pectinex Ultra SP-L enzyme was defined as the amount of enzyme that produced 1 µmol·min^–1^ of fructose.

### 2.3. Production of FOS Using Ultrasonication and Microbubbles

Brown sugar was dissolved in 0.1 M sodium acetate buffer (pH 6.5) with a final concentration of 70% (*w/v*). The solution (98 mL) was incubated at 55 °C for 1–2 h, then Pectinex Ultra SP-L was added with a final volume of 100 mL in a 250-mL Erlenmeyer flask. The reaction was performed at 55 °C with ultrasonication (37 kHz) using an ultrasonic bath (Elmasonic S 30 H, Elma Schmidbauer GmbH, Singen, Germeny) and then microbubbles were applied direct into the solution using a microbubble generator with a flow rate of 100 mL·min^–1^ and pressure of 0.25–0.5 mPa. When the reaction was stopped by incubation in boiling water, the FOS content in each sample was analysed using HPLC. For large scale production, the reaction was done in 1.5- and 5-L batches, and samples were collected every 1 h for 6 h. The FOS content in samples were analysed using HPLC and compared to a 100-mL scale.

### 2.4. Optimisation of FOS Production

In this study, a Box-Behnken experimental design with three levels of three factors and response surface methodology was used to estimate optimal levels of variables. The three factors with three levels for FOS production included ultrasonication time (*X*_1_, 2.00–15.00 min), microbubble time (*X*_2_, 5.00–30.00 min), and reaction time (*X*_3_, 2.00–6.00 h). Design expert 6.0.10 (Stat-Ease, Inc., Minneapolis, MN, USA) was used for experimental design and modelling analysis resulting in a total of 17 experiments, which were conducted in triplicate (Table 1). The model predicting the optimal values was expressed as Equation (1) given below:

(1) $$Y=\beta_{0}+\overset{k}{\underset{i=1}{\sum }}\beta_{i}X_{i}+\overset{k}{\underset{i=1}{\sum }}\beta_{ii}X_{i}^{2}+\overset{k}{\underset{i\geq j}{\sum }}\overset{k}{\underset{i=1}{\sum }}\beta_{ij}X_{ij}X_{j}$$

where *Y* is the predicted response, *β* is the regression coefficient and *X* is the independent variable. The *F*-value determined the statistical significance of the equation. The accuracy of the polynomial model equation was expressed by the coefficient of determination (*R*^2^).

### 2.5. Characterisation of FOS

The samples were filtered through a cellulose acetate membrane (0.22 μm; Sartorious, Göttingen, Germany) and then subjected to high-performance liquid chromatography (HPLC; HPLC 1260, Agilent Technology, Santa Clara, CA, USA) equipped with an Asahipak NH2P-50 4E column (5 μm, 250 × 4.6 mm; Showa Denko, Tokyo, Japan) and a refractive index (RI) detector. A mixture of acetonitrile and water (70:30) was used as a mobile phase at a flow rate of 1 mL·min^–^^1^ for 25 min. The column thermostat was 40 °C [36]. Peak identification and quantification of 1-kestose, nystose, 1^F^-*β*-fructofuranosylnystose, sucrose, glucose, and fructose in samples were estimated from the calibration curve, which was built with standard reference sugars (Wako Pure Chemical Industries, Osaka, Japan) under the same HPLC conditions.

### 2.6. Statistical Analysis 

All experiments were conducted in triplicate and the results were expressed as the mean ± standard deviations (SD). Statistical analysis of the results was performed using the SPSS statistical programme (version 17.0, IBM, Armonk, NY, USA). One-way analysis of variance (ANOVA), followed by Duncan’s multiple range test, was carried out, and the differences between individual means were assessed at *p* ≤ 0.05.

## 3. Results and Discussion

### 3.1. Production of FOS 

FOS were synthesised from brown sugar using a combination of ultrasonication and microbubbles, in which the Box-Behnken design was used. Actual values of enzyme activity and FOS yield (1-kestose, nystose, 1^F^-*β*-fructofuranosylnystose, and total FOS) obtained from an experimental design are shown in Table 1. Run 8 with 15 min of ultrasonication, 17.50 min of microbubbles and a 6.00 h reaction time led to the highest enzyme activity and a yield of 1^F^-*β*-fructofuranosylnystose and total FOS, which were 112.22 ± 2.50 U·mL^−1^, 24.61 ± 2.09 mg·g^−1^, and 525.45 ± 56.09 mg·g^−1^, respectively. Meanwhile, the highest yield of 1-kestose and nystose were obtained from Run 1 and Run 12, respectively, where the conditions were 2.00 min of ultrasonication, 5.00 min of microbubbles, and a 4.00 h reaction time for Run 1 and 8.50 min of ultrasonication, 30.00 min of microbubbles, and reaction time of 6.00 h for Run 12.

In addition, the relationship between the three variables (*X*_1_, *X*_2_ and *X*_3_) and the responses were analysed by ANOVA, which are shown in Table 2. According to the analysis, the reaction time (*X*_3_) was the most significant variable for Pectinex Ultra SP-L activity and production yield of 1-kestose, nystose, 1^F^-*β*-fructofuranosylnystose and total FOS. Moreover, the quadratic term, reaction time (*X*_3_^2^) displayed the most significant effect on enzyme activity and yield of 1-kestose and total FOS. However, there were no interactive effects and quadratic terms that had a significant effect on the yield of nystose and 1^F^-*β*-fructofuranosylnystose.

Considering the significant terms, enzyme activity and FOS yield could be described by the mathematical equations obtained in terms of coded variables. The predictive equations and statistical data are shown in Table 3. In the equations, a positive (+) coefficient indicates a synergistic effect, whereas a negative sign (–) refers to an antagonistic effect. According to the mathematical equations, the most significant variable was *X*_3_ (reaction time) because it had a positive effect on all responses. Moreover, *X*_1_ (ultrasonication time) also had a positive effect on the yield of nystose and 1^F^-*β*-fructofuranosylnystose. However, the interaction of the three variables showed both positive and negative effects. 

**Figure 1:**
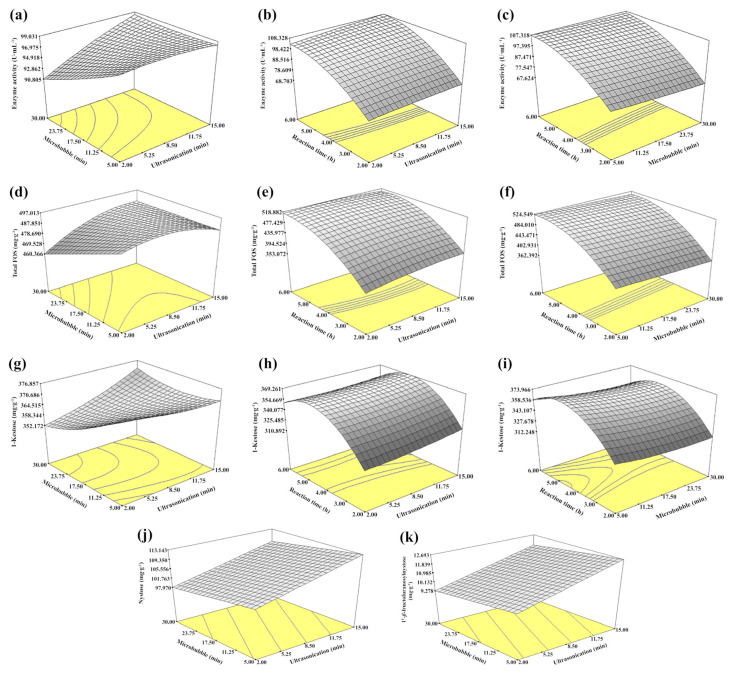
Response surface plots of the relationship between three variables (ultrasonication, microbubble and reaction time), (**a**–**c**) enzyme activity, (**d**–**f**) total FOS; (**g**–**i**) 1-kestose, (**j**) nystose, (**k**) 1^F^-*β*-fructofuranosylnystose.

The relationships between three variables and responses (enzyme activity, 1-kestose, nystose, 1^F^-*β*-fructofuranosylnystose and total FOS) are shown in Figure 1. The Pectinex Ultra SP-L activity (Figure 1a–c) and the yield of total FOS (Figure 1d–f) and 1-kestose (Figure 1g–i) increased when the reaction time (*X*_3_) was increased to 4–5 h. Moreover, ultrasonication time (*X*_1_) led to an increased yield of nystose (Figure 1j) and 1^F^-*β*-fructofuranosylnystose (Figure 1k), while microbubbles (*X*_2_) had no effect on them.

### 3.2. Optimisation of FOS Production

Based on the experimental designs, the optimum conditions for FOS production from brown sugar using ultrasonication and microbubbles were; (1) ultrasonication for 5 min 45 s, (2) microbubbles for 7 min 19 s, and (3) reaction time of 5 h 40 min. Under the optimum conditions, the predictive enzyme activity was 106.71 U·mL^–1^ and the predictive yield of kestose, nystose, 1^F^-*β*-fructofuranosylnystose and total FOS were 365.25, 147.92, 17.92 and 525.44 mg·g^–1^, respectively. To validate the optimum conditions, validation experiments were performed using white sugar and brown sugar as substrates. The actual values obtained from experiments under each set of conditions are shown in Table 4. The combination of ultrasonication and microbubbles enhanced the Pectinex Ultra SP-L activity up to 102.51 ± 4.69 U·mL^–1^, which produced a total FOS yield of 494.89 ± 19.98 mg·g^−1^ substrate using brown sugar. For white sugar, the enzyme activity was 107.93 ± 5.26 U·mL^–1^ and total FOS yield was increased to 580.01 ± 21.35 mg·g^−1^ substrate. When the ultrasonication was performed without microbubbles, the enzyme activity and total FOS yield were decreased. This result confirmed that a microbubble step was important for the production. According to a previous report, Pectinex Ultra SP-L activity under normal conditions was 22 U·mL^–1^ [5]. Therefore, Pectinex Ultra SP-L activity was increased by 366% and 390% for brown sugar and white sugar, respectively, when the combined ultrasonication and microbubble were performed. Noticeably, white sugar led to a higher enzyme activity and total FOS yield than brown sugar (Table 4). Thus, white sugar was appropriate to use as a substrate for FOS production. Brown sugar contains sucrose, glucose, and fructose. Glucose in brown sugar could inhibit fructosyltransferase activity via feedback inhibition, so total FOS yield was lower [21]. According to the validation, the error was 5.18% between the predicted model and experimental values. Hu [37] reported that the error value should be less than 10%, so the model was acceptable to predict the production of FOS using Pectinex Ultra SP-L with a combination of ultrasonication and microbubbles.

### 3.3. Production of FOS in a Large Scale

**Figure 2:**
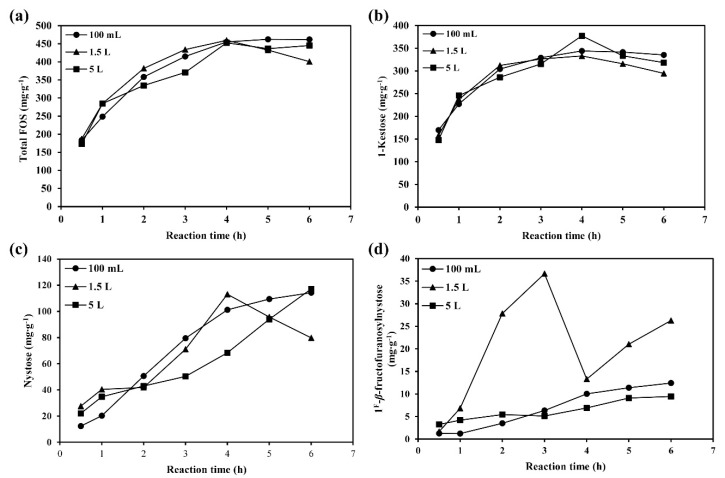
Fructooligosaccharide production from brown sugar in 100 mL, 1.5 L and 5 L batches. (**a**) Total FOS yield, (**b**) 1-Kestose yield, (**c**) Nystose yield, and (**d**) 1^F^-*β*-fructofuranosylnystose yield.

The scale-up of FOS production was performed at 0.1, 1.5 and 5.0-L scales under the optimum conditions described above. The samples were collected every 1 h for a total of 6 h (Figure 2. At the 100-mL scale, the maximum yield of 1-kestose, nystose, 1^F^-*β*-fructofuranosylnystose, and total FOS were obtained at 4, 6, 6, and 5 h reaction time, respectively. When a volume of 1.5-L production was used, the optimum reaction time was 4, 4, 3 and 4 h for 1-kestose, nystose, 1^F^-*β*-fructofuranosyl-nystose, and total FOS, respectively. While the maximum yield of 1-kestose, nystose, 1^F^-*β*-fructofuranosylnystose, and total FOS at the 5-L scale occurred at 4, 6, 6 and 4 h reaction time, respectively. In the 100-mL batch, the highest total FOS yield was 462.34 ± 24.22 mg·g^−1^ substrate at a 5 h reaction. While the optimum reaction time of the enlarge production scale (1.5 and 5 L) decreased to 4 h, the maximum yields of total FOS were 459.24 ± 25.64 and 452.55 ± 19.53 mg·g^−1^ substrate, respectively (Figure 2a). Thus, the enlarged production took a short reaction time than 100-mL production. Interestingly, the FOS yields produced in a 1.5-L batch were much higher than other batches at the optimum reaction time (Figure 2a,c,d). Therefore, 1.5-L scale might be appropriate for FOS production from brown sugar and the enlarged production scale decreased the reaction time, compared to the 100-mL scale. 

In comparison, the total FOS yield from brown sugar was 0.45 g·g^−1^ substrate, which was lower than that of white sugar (0.58 g·g^−1^ substrate). Despite a low FOS yield, brown sugar had a shorter reaction time (4 h) than white sugar (5.67 h). Table 5 presents the total FOS yield obtained from this study in comparison to other studies. In previous studies, they used the conventional method and ultra-high pressure (UHP)-assisted method to synthesise FOS from sugar. The UHP.assisted method took the shortest reaction time (15 min) and had a total FOS yield of 0.57 g·g^−1^ substrate [5]. Among the conventional method, FOS yield ranged from 0.55 to 0.67 g·g^−1^ substrate within a reaction time of 5.6 to 16 h [38,39,40,41,42,43]. Although the FOS yield from this study was lower, the reaction time was reduced. The normal reaction time in the conventional method was ~10 h when using Pectinex Ultra SP-L in the synthesis. Here we found that the use of ultrasonication and microbubbles could decrease reaction time by 60%.

This study demonstrated that the combination of ultrasonication and microbubbles could enhance FOS concentration and productivity compared to the conventional method (Table 6). In 4 h of reaction time, the combined methods gave the maximum concentration of 1-kestose (264.1 g·L^−1^) and total FOS (316.8 g·L^−1^), which also had the highest productivity (66.0 and 79.2 g·L^−1^·h^–1^, respectively). Conversely, the concentration and productivity of nystose and 1^F^-*β*-fructofuronosyl nystose were lower than in previous studies. This confirmed that using the combined methods had a higher productivity of FOS from brown sugar, despite a low FOS yield. Although the UHP-assisted approach produced the highest productivity of FOS, this method required complex high-pressure equipment systems and consumed a lot of energy due to extreme pressure [5]. In contrast, the ultrasonication and microbubble techniques were simple methods with mild conditions that consumed less energy. Additionally, these techniques have been accepted as cost-effective and eco-friendly processes [28]. Consequently, this method was suitable to apply on an industrial scale for FOS production. Many studies have reported that ultrasound at an appropriate frequency led to changes in enzyme conformation. The conformational changes had important roles in catalysis. A new conformation might enhance binding with substrates, therefore increasing enzyme activity [28,29,44].

With regard to microbubbles, the effect of microbubbles on enzymes and the production is still unclear and requires further investigation. Microbubbles might create turbulence in the system and increase the possibility of enzyme-substrate attachment. Based on the increased surface area of microbubbles, we speculated that microbubbles might adsorb sugar molecules on their surfaces and carry them to the catalytic sites of enzymes, thereby increasing the activity [45]. Furthermore, various reports have confirmed that high pressure has both positive and negative effects on enzyme activity. Adequate pressure can enhance enzyme activity [46], for the reason that microbubbles are unstable, are suddenly broken and gas is released. Spontaneously, pressure is generated and affects the enzyme. We suggested that the pressure from microbubbles affected enzyme activity, so FOS yields and enzyme activity were increased.

We found that the technique is useful and easy to use for FOS production and has the potential to be applied in industrial production. In the future, the combination of ultrasonication and microbubbles has the potential to increase production of other oligosaccharides, including galactooligosaccharides (GOS), xylooligosaccharides (XOS), inulooligosaccharides (IOS), and soybean oligosaccharides (SOS) and should be considered in future research.

## 4. Conclusions

The optimal conditions for FOS production from brown sugar were the ultrasonication for 5 min 45 s, microbubbles for 7 min 19 s, and a reaction time of 5 h 40 min. The combination of ultrasonication and microbubbles could enhance enzyme activity, and the Pectinex Ultra SP-L activity reached 102.51 ± 4.69 U·mL^−1^. The highest yield of total FOS, 1-kestose, nystose, and 1^F^-*β*-fructofuranosylnystose were 494.89 ± 19.98, 368.48 ± 20.22, 110.53 ± 8.81, and 16.35 ± 1.49 mg·g^−1^ substrate, respectively. On a large scale (5 L), the maximum yield of total FOS, 1-kestose, nystose, and 1^F^-*β*-fructofuranosylnystose were 452.55 ± 19.53, 377.28 ± 21.64, 68.35 ± 2.35 and 6.92 ± 0.09 mg·g^−1^ substrate, respectively within 4 h of reaction. Thus, the use of ultrasonication and microbubble in FOS production using brown sugar was able to increase Pectinex Ultra SP-L activity by 366% and reduce the reaction time by 60%, which productivity of FOS was enhanced. 

This strategy has various advantages, such as easy and safe performance, and high efficacy. Additionally, this study uses a low-cost substrate (brown sugar) in which significantly reduces production costs and affects the economy of the process. Therefore, the use of brown sugar as a substrate with ultrasonication and microbubble assistants is an appropriate strategy for FOS production at an industrial scale.

## Figures and Tables

**Table 1:** The Box-Behnken experimental design and experimental values of enzymatic activity and FOS yields produced from brown sugar.

Run	Variable			Enzyme Activity (U·mL^–1^)	Oligosaccharide Yields (mg·g^–1^)			
	*X*_1_ (min) *	*X*_2_ (min) **	*X*_3_ (h) ***		1-Kestose	Nystose	1^F^-*β*-Fructofuranosylnystose	Total FOS
1	2.00	5.00	4.00	102.03 ± 1.88	379.80 ± 23.15	112.84 ± 1.55	10.30 ± 0.06	502.94 ± 35.46
2	15.00	5.00	4.00	97.75 ± 4.10	371.04 ± 34.06	108.94 ± 2.33	12.24 ± 0.15	492.22 ± 26.12
3	2.00	30.00	4.00	91.44 ± 2.30	351.19 ± 28.85	95.27 ± 1.69	8.31 ± 0.25	454.77 ± 44.59
4	15.00	30.00	4.00	95.13 ± 4.50	362.96 ± 49.73	103.95 ± 2.03	9.14 ± 0.59	476.05 ± 36.21
5	2.00	17.50	2.00	64.82 ± 0.98	311.30 ± 32.11	41.36 ± 0.96	2.09 ± 0.06	354.74 ± 25.89
6	15.00	17.50	2.00	70.88 ± 2.10	330.06 ± 44.53	52.42 ± 1.12	3.02 ± 0.12	385.50 ± 38.01
7	2.00	17.50	6.00	102.67 ± 2.71	341.32 ± 25.85	146.26 ± 3.54	18.65 ± 1.06	506.26 ± 46.04
8	15.00	17.50	6.00	112.22 ± 2.50	352.25 ± 25.40	148.59 ± 3.59	24.61 ± 2.09	525.45 ± 56.09
9	8.50	5.00	2.00	73.95 ± 2.59	313.25 ± 35.33	55.56 ± 0.58	2.96 ± 0.08	371.77 ± 34.56
10	8.50	30.00	2.00	70.87 ± 3.50	313.25 ± 27.56	50.43 ± 0.49	2.65 ± 0.11	366.32 ± 23.15
11	8.50	5.00	6.00	104.07 ± 3.15	358.53 ± 16.81	143.10 ± 4.32	17.28 ± 0.56	518.92 ± 35.11
12	8.50	30.00	6.00	105.04 ± 4.97	356.39 ± 40.74	149.94 ± 2.51	18.67 ± 0.92	525.00 ± 43.65
13	8.50	17.50	4.00	94.47 ± 1.39	367.94 ± 32.00	100.38 ± 2.09	8.32 ± 0.49	476.64 ± 35.28
14	8.50	17.50	4.00	99.41 ± 6.83	361.44 ± 42.75	120.14 ± 3.05	12.75 ± 0.46	494.33 ± 43.19
15	8.50	17.50	4.00	95.86 ± 2.11	354.56 ± 41.56	111.06 ± 1.54	10.62 ± 0.54	476.24 ± 54.56
16	8.50	17.50	4.00	95.60 ± 3.25	365.72 ± 26.35	109.90 ± 4.56	12.26 ± 0.83	487.89 ± 35.21
17	8.50	17.50	4.00	98.66 ± 2.17	359.31 ± 25.50	122.59 ± 3.97	12.89 ± 0.49	494.79 ± 43.54

* *X*_1_, ultrasonication time; ** *X*_2_, Microbubble time; *** *X*_3_, Reaction time.

**Table 2:** Analysis of variance (ANOVA) for the response surface quadratic model for FOS production.

	Enzyme Activity		1-Kestose		Nystose		1^F^-*β*-Fructofuranosylnystose		Total FOS	
	*F* Value	*p* Value	*F* Value	*p* Value	*F* Value	*p* Value	*F* Value	*p* Value	*F* Value	*p* Value
Model	24.57	0.0002	8.64	0.0048	75.78	<0.0001	54.54	<0.0001	42.45	<0.0001
*X*_1_ *	2.10	0.1904	0.985	0.354	2.13	0.168	3.18	0.0979	1.58	0.248
*X*_2_ **	2.18	0.1832	2.87	0.134	0.573	0.462	0.545	0.473	3.78	0.0931
*X*_3_ ***	191.53	<0.0001	27.92	0.0011	224.63	<0.0001	159.89	<0.0001	310.96	<0.0001
*X*_1_ *X*_2_	1.18	0.3135	2.03	0.197					1.91	0.210
*X*_1_ *X*_3_	0.225	0.6494	0.173	0.690					1.76	0.226
*X*_2_ *X*_3_	0.304	0.5984	0.0128	0.913					0.247	0.634
*X*_1_^2^	0.0859	0.7780	0.230	0.646					1.66	0.239
*X*_2_^2^	0.0307	0.8660	0.694	0.432					0.00004	0.995
*X*_3_^2^	23.34	0.0019	43.55	0.0003					58.68	0.0001
Lack of Fit	5.61	0.0645	6.06	0.0572	1.27	0.438	0.981	0.553	4.34	0.0951

* *X*_1_, ultrasonication time (min); ** *X*_2_, Microbubble time (min); *** *X*_3_, Reaction time (h).

**Table 3:** Analysis of variance (ANOVA) of the model and coefficient estimates for response parameters.

Response	Final Equation in Term of Actual Factors	*P* *	LOF	*R* ^2^	Adj. *R*^2^	AP	CV
Enzyme activity	35.10–0.20 × *X*_1_–0.59 × *X*_2_ + 24.95 × *X*_3_–0.012 × *X*_1_^2^ + 0.002 × *X*_2_^2^–2.16 × *X*_3_^2^ + 0.024 × *X*_1_ × *X*_2_ + 0.07 × *X*_1_ × *X*_3_ + 0.04 × *X*_2_ × *X*_3_	0.0002	0.0645	0.9693	0.9299	14.480	3.96
1-Kestose	226.07–1.22 × *X*_1_–1.92 × *X*_2_ + 70.97 × *X*_3_ + 0.01 × *X*_1_^2^ + 0.02 × *X*_2_^2^–7.57 × *X*_3_^2^ + 0.08 × *X*_1_ ×*X*_2_–0.15 × *X*_1_ ×*X*_3_–0.02 × *X*_2_ × *X*_3_	0.0048	0.0572	0.9175	0.8113	9.140	2.69
Nystose	12141 + 0.77 × *X*_1_–0.21 × *X*_2_ + 25.63 × *X*_3_	<0.0001	0.4385	0.9459	0.9334	23.978	9.17
1^F^-*β*-Fructofuranosyl-nystose	–7.02 + 0.19 × *X*_1_–0.04 × *X*_2_ + 4.28 × *X*_3_	<0.0001	0.5529	0.9264	0.9094	21.032	17.44
Total FOS	165.75 + 4.35 × *X*_1_–1.94 × *X*_2_ + 125.68 × *X*_3_–0.17 × *X*_1_^2^ + 0.0002 × *X*_2_^2^–10.82 × *X*_3_^2^ + 0.10 × *X*_1_ × *X*_2_–0.59 × *X*_1_ × *X*_3_ + 0.12 × *X*_2_ × *X*_3_	<0.0001	0.0951	0.9820	0.9589	19.098	2.49

*P, probability of error; LOF, lack of fit; Adj. *R*^2^, adjust *R*^2^; AP, adequate precision; CV, coefficient of variance.

**Table 4:** Fructooligosaccharide (FOS) yields produced under optimal conditions: (*X*_1_) ultrasonication 5 min 45 s, (*X*_2_) microbubble 7 min 19 s, and (*X*_3_) reaction time of 5 h 40 min.

Factors	Pectinex Ultra SP-L Activity (U·mL^−1^)	Fructooligosaccharide Yields (mg·g^−1^)			
		1-Kestose	Nystose	1^F^-*β*-Fructofurano-sylnystose	Total FOS
Brown sugar using a combination of ultrasonication and microbubble	102.51 *±* 4.69	368.02 *±* 20.22	110.53 *±* 8.81	16.35 *±* 1.49	494.89 *±* 19.98
White sugar using a combination of ultrasonication and microbubbles	107.93 *±* 5.26	415.02 *±* 19.54	147.50 *±* 9.84	17.49 *±* 2.94	580.01 *±* 21.35
White sugar using only ultrasonication	90.05 *±* 3.54	399.48 *±* 21.33	103.83 *±* 12.33	10.23 *±* 1.66	515.53 *±* 20.43

**Table 5:** Comparison of FOS production from this study and other reports.

Substrate	Enzyme	Condition	Reaction Time (h)	FOS Yield (g·g^−1^ Substrate)	Reference
Brown sugar	Pectinex Ultra SP-L	Ultrasonication and microbubble, 55 °C, 700 g·L^−1^ substrate	4	0.45	This study
White sugar	Pectinex Ultra SP-L	Ultrasonication and microbubble, 55 °C, 700 g·L^−1^ substrate	5.67	0.58	This study
White sugar	GAPfopA_V1 (engineered *β*-fructofuronosidase)	Water bath, 62 °C, 120 rpm, 600 g·L^−1^ substrate	5. 6	0.55	[43]
White sugar	Pectinex Ultra SP-L	Ultra-high pressure at 300 MPa, 30 °C, 600 g·L^−1^ substrate	0.25	0.57	[5]
White sugar and Jerusalem artichoke	Inulinase	Water bath, 55 °C, 59.4 g·L^−1^ inulin and 598.7 g·L^−1^ refine sugar	9	0.67	[38]
White sugar	Pectinex Ultra SP-L	Water bath, 60 °C, 400 g·L^−1^ substrate	16	0.62	[39]
White sugar	Pectinex Ultra SP-L	Water bath, 55 °C, 500 g·L^−1^ substrate	10	0.60	[40]
White sugar	Pectinex Ultra SP-L	Water bath, 50 °C, 536.2 g·L^−1^ substrate	6	0.59	[41]
White sugar	Culture fluid of *Aspergillus oryzae* CFR202	Water bath, 55 °C, 64.55 g·L^−1^ substrate	12	0.58	[42]

**Table 6:** Fructooligosaccharide (FOS) yield and productivity obtained from this study compared to other reports.

Method	Reaction Time (h)	Fructooligosaccharide								Reference
		Concentration (g·L^−1^)				Productivity (g·L^−1^·h^−1^)				
		GF_2_ *	GF_3_	GF_4_	Total FOS	GF_2_	GF_3_	GF_4_	Total FOS	
Ultrasonic and microbubble methods	4	264.1	47.8	4.84	316.8	66.0	11.96	1.21	79.2	This study
Ultra-high pressure (UHP) method	0.25	189.72	110.88	28.2	328.8	758.88	443.52	112.8	1315.2	[5]
Conventional methods	5.6	220.8	321.6	60.0	330	39.4	57.4	10.7	58.9	[43]
	9	156.98	144.10	84.63	404.0	17.44	16.01	9.40	44.90	[38]
	10	155	130	15.0	300	15.5	13.0	1.50	30.0	[40]
	6	212.33	106.7	8.58	327.6	35.4	17.78	1.43	54.6	[41]
	12	174.2	96.6	26.8	297.7	14.5	8.05	2.24	24.8	[42]

* GF_2_, 1-Kestose; GF_3_, Nystose; GF_4_, 1^F^-*β*-fructofuronosylnystose.
